# Structural changes in amygdala nuclei, hippocampal subfields and cortical thickness following electroconvulsive therapy in treatment-resistant depression: longitudinal analysis

**DOI:** 10.1192/bjp.2018.224

**Published:** 2019-03

**Authors:** Gregor Gryglewski, Pia Baldinger-Melich, René Seiger, Godber Mathis Godbersen, Paul Michenthaler, Manfred Klöbl, Benjamin Spurny, Alexander Kautzky, Thomas Vanicek, Siegfried Kasper, Richard Frey, Rupert Lanzenberger

**Affiliations:** 1 Resident, Department of Psychiatry and Psychotherapy, Medical University of Vienna, Austria; 2 Consultant Psychiatrist, Department of Psychiatry and Psychotherapy, Medical University of Vienna, Austria; 3 Research Associate, Department of Psychiatry and Psychotherapy, Medical University of Vienna, Austria; 4 Resident, Department of Psychiatry and Psychotherapy, Medical University of Vienna, Austria; 5 Resident, Department of Psychiatry and Psychotherapy, Medical University of Vienna, Austria; 6 Research Assistant, Department of Psychiatry and Psychotherapy, Medical University of Vienna, Austria; 7 Research Assistant, Department of Psychiatry and Psychotherapy, Medical University of Vienna, Austria; 8 Resident, Department of Psychiatry and Psychotherapy, Medical University of Vienna, Austria; 9 Resident, Department of Psychiatry and Psychotherapy, Medical University of Vienna, Austria; 10 Chair, Department of Psychiatry and Psychotherapy, Medical University of Vienna, Austria; 11 Vice Chair, Department of Psychiatry and Psychotherapy, Medical University of Vienna, Austria; 12 Associate Professor and Head of the Neuroimaging Labs, Department of Psychiatry and Psychotherapy, Medical University of Vienna, Austria

**Keywords:** Electroconvulsive therapy, imaging, inpatient treatment, depressive disorders

## Abstract

**Background:**

Electroconvulsive therapy (ECT) is the treatment of choice for severe mental illness including treatment-resistant depression (TRD). Increases in volume of the hippocampus and amygdala following ECT have consistently been reported.

**Aims:**

To investigate neuroplastic changes after ECT in specific hippocampal subfields and amygdala nuclei using high-resolution structural magnetic resonance imaging (MRI) (trial registration: clinicaltrials.gov – NCT02379767).

**Method:**

MRI scans were carried out in 14 patients (11 women, 46.9 years (s.d. = 8.1)) with unipolar TRD twice before and once after a series of right unilateral ECT in a pre–post study design. Volumes of subcortical structures, including subfields of the hippocampus and amygdala, and cortical thickness were extracted using FreeSurfer. The effect of ECT was tested using repeated-measures ANOVA. Correlations of imaging and clinical parameters were explored.

**Results:**

Increases in volume of the right hippocampus by 139.4 mm^3^ (s.d. = 34.9), right amygdala by 82.3 mm^3^ (s.d. = 43.9) and right putamen by 73.9 mm^3^ (s.d. = 77.0) were observed. These changes were localised in the basal and lateral nuclei, and the corticoamygdaloid transition area of the amygdala, the hippocampal–amygdaloid transition area and the granule cell and molecular layer of the dentate gyrus. Cortical thickness increased in the temporal, parietal and insular cortices of the right hemisphere.

**Conclusions:**

Following ECT structural changes were observed in hippocampal subfields and amygdala nuclei that are specifically implicated in the pathophysiology of depression and stress-related disorders and retain a high potential for neuroplasticity in adulthood.

**Declaration of interest:**

S.K. has received grants/research support, consulting fees and/or honoraria within the past 3 years from Angelini, AOP Orphan Pharmaceuticals AG, AstraZeneca, Celegne GmbH, Eli Lilly, Janssen-Cilag Pharma GmbH, KRKA-Pharma, Lundbeck A/S, Neuraxpharm, Pfizer, Pierre Fabre, Schwabe and Servier. R.L. received travel grants and/or conference speaker honoraria from Shire, AstraZeneca, Lundbeck A/S, Dr. Willmar Schwabe GmbH, Orphan Pharmaceuticals AG, Janssen-Cilag Pharma GmbH, and Roche Austria GmbH.

To neuroscientists in clinical practice the hitherto unsurpassed efficacy of electroconvulsive therapy (ECT) in the treatment of severe mental illness, such as catatonia, treatment-resistant depression (TRD) or clozapine-resistant psychosis,^1^ is a daily reminder of the importance of unveiling its mechanisms of action. The measurement of changes in brain structure using magnetic resonance imaging (MRI) allows for the investigation of neuroplastic processes during ECT. Despite increases in volume of the hippocampus and amygdala following ECT being among the most consistently reported effects in psychiatric neuroimaging, no reproducible association of structural MRI outcomes and depressive symptoms has been established so far.^2^^–^^4^ However, an analysis of hippocampal subfields and nuclei of amygdala was not undertaken in most previous studies. which were performed before the emergence of novel segmentation techniques.^5^^,^^6^

In contrast, a recent study that scrutinised the effects of bitemporal ECT on hippocampal subfields in relation to its efficacy reported significant increases in the bilateral cornu ammonis 4 and granule cell layer of the dentate gyrus, and implicated baseline differences in several subfields as predictors of antidepressant response.^9^ These results align with the finding of reductions in dentate gyrus volumes being associated with the number of depressive episodes^10^ and lower cornu ammonis and dentate gyrus volumes in unmedicated patients with depression compared with medicated patients and healthy individuals.^11^ More support for these subfields as high potential biomarkers can be derived from reported reductions in cornu ammonis 2–3, cornu ammonis 4 and dentate gyrus in subjects scoring high on questionnaires for traumatic life events.^12^ The study of alterations of the amygdala in major depression and during treatment was prominently focused on functional differences in activation during emotional processing.^13^ Reported effects of depression on the volume of the structure were less consistent, and systematic assessment of nuclei of the amygdala was hampered by the lack of reliable automated segmentation pipelines for analysis of human imaging data.^5^ Still, all published studies investigating the volume of the amygdala after ECT reported increases.^3^^,^^4^^,^^14^ In the light of these findings, we aimed to investigate changes in hippocampal subfield volumes in patients with TRD undergoing ECT with right unilateral stimulation. Furthermore, we use a recently developed segmentation pipeline to disentangle the effect of ECT on specific nuclei of the amygdala.^5^ Finally, we comprehensively report changes in cortical thickness observed following ECT.

## Method

### Participants and study design

Individuals experiencing a severe TRD episode were included in this interventional pre–post study. All patients received open treatment with an ECT series consisting of a minimum of eight right unilateral sessions between baseline and follow-up assessments. Recruitment was performed at the in-patient clinic of the Department of Psychiatry and Psychotherapy at the Medical University of Vienna. Eligible patients were adults (18–60 years) diagnosed with a severe major depressive episode according to ICD-10 with or without psychotic symptoms (F32.2, F32.3, F33.2, F33.3)^15^ scheduled for ECT treatment. Patients with a diagnosis of bipolar affective disorder, schizoaffective disorder or schizophrenia were excluded from participation. Furthermore, patients with current substance misuse, including nicotine, were not included in the trial. Clinical diagnoses by senior psychiatrists were supported by structured clinical interviews for DSM-IV (SCID-I).^16^ Based on retrospective assessments of recorded data, criteria for diagnosis of major depressive disorder according to DSM-5 were fulfilled by all the included patients.^17^ A 17-item Hamilton Rating Scale for Depression (HRSD) score of 23 or higher was required for enrolment of patients.^18^

All patients had undergone prior treatment with a minimum of two antidepressants (adequate dosage according to prescribing information) or augmentative psychopharmacological agents in the current episode. Patients were required to be on stable medication for a minimum of 10 days leading up to the baseline assessments to minimise variance. Rescue medication with benzodiazepines was allowed. Patients who had undergone ECT in the past were not considered for participation. Patients had to have been free from medication with monoamine oxidase inhibitors in the 6 months prior to participation.

General health was assessed with a physical examination, electrocardiography, thoracic X-ray and routine laboratory tests (including thyroid function, blood cell counts, electrolytes and virology). Clinically relevant deviations in laboratory parameters led to exclusion of patients. Patients with contraindications for MRI scans were also excluded from participation. Urine tests were carried out to screen for pregnancy and substance misuse (cotinine, tetrahydrocannabinol, opioids, amphetamines, benzodiazepines, barbiturates, cocaine, methamphetamine, methadone, tricyclic antidepressants, methylenedioxymethamphetamine). All patients provided written informed consent. All study procedures were carried out according to good clinical practice guidelines and the Declaration of Helsinki and approved by the ethics committee of the Medical University of Vienna. The study was registered before the beginning of recruitment at clinicaltrials.gov (NCT02379767).

### ECT treatment

Each patient received a minimum of eight right unilateral ECT sessions according to standard operating procedures implemented at the Department of Psychiatry and Psychotherapy of the Medical University of Vienna. Additional ECT sessions were performed to assure continuous therapy in case follow-up assessments were delayed because of technical issues. Right unilateral treatment was maintained in all patients during participation in the trial to avoid variability because of changes in electrode placement. ECT treatments were carried out using the Thymatron System IV device (Somatics, LLC, Lake Bluff, Illinois, USA). Sessions were performed on working days three times a week at most. The first stimulation dose in each patient was set to 50 mC (10%) and was applied using an ultra-brief pulse width (0.25 ms).

Electroencephalography, electrocardiography and an electromyogram of one forearm were recorded during seizures. The stimulation resulted in an adequate seizure based on duration, amplitude, ictal coherence and post-ictal suppression in each patient. From the second session onwards stimulation was performed with thrice the seizure threshold and was monotonically increased in case of inadequate seizure quality. On the days of treatment ongoing medication with psychopharmacological drugs potentially affecting seizure threshold and increasing blood pressure was administered after ECT to reduce the chance of adverse effects. Anaesthesia was performed using methohexital and the muscle relaxant succinylcholine.

### MRI and data processing

MRI was carried out on a 3Tesla PRISMA MR Scanner (Siemens Medical, Erlangen, Germany) using a 64-channel head coil to acquire *T*_1_-weighted structural data. Patients were scanned twice before ECT treatment (separated by 1–5 days) in order to obtain a solid baseline and estimate robustness of outcome measures, and once after ECT. Post-ECT MRI was performed at least 24 h after the last treatment. An MPRAGE sequence (repetition time/echo time 2000/2.9 ms) with an isotropic voxel size 1 × 1 × 1 mm (200 slices) was acquired. Cortical surface reconstruction and parcellation of subcortical regions was performed automatically using the recon-all pipeline in FreeSurfer 6.0 with standard parameters (Harvard Medical School, Boston, USA; http://surfer.nmr.mgh.harvard.edu/). Furthermore, segmentation of hippocampal subfields and amygdala nuclei was carried out to scrutinise the effect of ECT in specific substructures of these regions.^5^^,^^6^ In the longitudinal processing pipeline a within-participant template created from all time points using inverse consistent registration was used for skull stripping, Talairach registration and initialisation of cortical surface reconstruction, cortical atlas registration and subcortical parcellation. Volumes of subcortical regions and mean cortical thickness (distance between pial and white matter surface) for regions of the Desikan Killiany atlas^7^ at each time point were extracted for statistical analysis.

### Statistical analysis

Test–retest reliability of outcome measures for the two MRI scans performed before ECT was assessed as described previously.^8^ Intraclass correlation coefficients (ICC) were calculated for each region using ICC1 in the psych package in R3.5.0. Furthermore, mean change and variability were calculated as the mean and standard deviation of the relative difference between the two scans. Based on the high ICC of imaging outcome measures, data from both scans prior to ECT was averaged and compared with post-ECT scans. The effect of ECT on volumes of subcortical regions and cortical thickness was assessed using repeated-measures ANOVA in SPSS 25.0. Mauchly's sphericity tests were performed to exclude deviations from the sphericity assumption. Two-way ANOVAs were constructed to test the within-participant effects of time point (pre-ECT (MRI 1 and MRI 2 averaged) versus post-ECT MRI) and brain region, and their interaction.

ANOVAs were separately performed for subcortical structures and cortical thickness of each hemisphere, respectively, based on the expectation of a lateralisation of ECT effects.^2^^,^^3^ Based on significant effects in the right amygdala and right hippocampus, the effect of ECT in subfields of the right hippocampus and nuclei of the right amygdala were tested. To assess the effect of ECT in each sub-/region post-hoc testing was performed with conservative Bonferroni correction for family-wise error (FWE) by multiplying *P*-values with the number of regions (7 for subcortical regions, 28 for subfields of the right hippocampus and nuclei of the right amygdala, 34 for cortical areas). Results were considered significant at an FWE-corrected probability of type I error of α ≤ 0.05. Additionally, trend-level effects were reported after controlling for false discovery rate (FDR) using the Benjamini–Hochberg procedure at a level of q* ≤ 0.05. Effect sizes are further listed as absolute and relative mean differences between an average of baseline values and post-ECT outcome measures.

Aiming to replicate results by Cao *et al*,^9^ an explorative analysis was carried out by calculating Pearson correlation coefficients between the relative change in HRSD scores during study participation and the volumes of subcortical structures, amygdala nuclei, hippocampal subfields and cortical thickness pre-ECT and their change after ECT, respectively. Results with an uncorrected *P*-value below 0.01 were reported.

## Results

### Clinical data and availability of MRI data

In total, 16 patients were enrolled in the trial. One patient dropped out because of a phobic reaction in the scanner and one patient was not included in the current analysis as MRI was performed on a different scanner. The final 14 patients (3 men) were aged 46.9 years (s.d. = 8.1), 13 had recurrent depressive disorder and 3 had psychotic symptoms. HRSD scores at baseline were 25.4 (s.d. = 3.3) and 7.1 (s.d. = 4.2) at study completion. In nine patients, depressive symptoms remitted with a HRSD ≤7 and only one patient did not respond to ECT (HRSD change below 50%).

A maximum of 11 ECT sessions were performed (8.6 on average) before post-ECT MRI. Average seizure duration was 41.4 s (s.d. = 0.6). Clinical data for individual participants including medication is shown in Table 1. Data of one of the two baseline MRI scans is missing in two patients. For one patient, post-ECT MRI data are missing, but baseline data were included in analysis. In one patient, subcortical segmentation in one baseline scan showed a marked deviation (areas of the right hippocampus were not identified correctly rendering it more than 500 mm^3^ smaller compared with other scans from the participant) and was excluded from analysis. In one patient, subcortical segmentation of the post-ECT MRI data had to be excluded because of segmentation of choroidal plexus within the right hippocampus, which did not occur in baseline data, which rendered it an outlier (more than 200 mm^3^ smaller compared with pre-ECT).

**Table 1:** Clinical and demographical data of individual patients included in analysis

Participant	MRI data	ICD-10 diagnosis	Age, years	Gender	HRSD baseline	HRSD post-ECT	ECT sessions	Mean seizure duration, s	Maximum stimulus, %	Continued psychopharmacological medication		
										Antidepressants	Augmentation	Sedatives/anxiolytics
1	Post-ECT MRI missing	F33.3	47	Woman	25	10	8	24.9	100	duloxetine 120 mg trazodone 300 mg	aripiprazole 30 mg risperidone 2 mg	lorazepam 5 mg levomepromazine 25 mg
2	2nd baseline MRI missing	F33.2	50	Woman	24	5	8	32.0	100	venlafaxine 300 mg amitriptyline 100 mg	aripiprazole 20 mg	prothipendyl 80 mg
3	Complete	F33.2	40	Man	23	11	8	59.4	80	venlafaxine 300 mg mirtazapine 60 mg	quetiapine 75 mg	clonazepam 1.5 mg
4	Complete	F33.2	55	Woman	23	7	8	56.3	50	vortioxetine 40 mg	quetiapine 300 mg lamotrigine 50 mg	clonazepam 0.5 mg
5	Complete	F33.3	43	Woman	28	10	8	38.3	100	venlafaxine 225 mg trazodone 150 mg	quetiapine 400 mg risperidone 4 mg	–
6	Complete	F33.2	42	Man	23	16	10	33.9	200	escitalopram 20 mg	lamotrigine 200 mg	–
7	Complete	F33.2	56	Woman	23	1	10	38.4	150	vortioxetine 10 mg escitalopram 30 mg venlafaxine 225 mg	lamotrigine 100 mg	lorazepam 3 mg
8	Subcortical data in post-ECT MRI excluded	F33.2	53	Woman	33	4	9	26.7	150	fluoxetine 40 mg mianserin 60 mg	–	clonazepam 2 mg pregabalin 250 mg prothipendyl 80 mg
9	Complete	F32.2	41	Woman	31	5	8	31.9	100	sertraline 150 mg trazodone 150 mg	risperidone 0.5 mg	clonazepam 1 mg
10	Complete	F33.2	33	Woman	22	5	8	44.6	40	sertraline 150 mg	aripiprazole 5 mg	lorazepam 1.5 mg
11	Complete	F33.2	49	Woman	23	7	11	43.1	100	vortioxetine 20 mg trazodone 50 mg	aripiprazole 5 mg	clonazepam 0.25 mg
12	Complete	F33.2	35	Man	24	12	8	55.5	50	escitalopram 20 mg	quetiapine 50 mg	–
13	2nd baseline MRI missing	F33.2	58	Woman	27	3	8	49.9	100	venlafaxine 300 mg mirtazapine 30 mg	aripiprazole 5 mg quetiapine 50 mg	lorazepam 2.5 mg
14	Subcortical data in 2nd baseline MRI excluded	F33.3	55	Woman	26	3	8	44.4	50	sertraline 150 mg, mirtazapine 45 mg	aripiprazole 15 mg	lorazepam 5 mg

MRI, magnetic resonance imaging; HRSD, Hamilton Rating Scale for Depression; ECT, electroconvulsive therapy.

### Test–retest reliability of imaging parameters at baseline

Data from both MRI scans acquired before ECT was available for 12 participants, and data from 11 participants was available for analysis of test–retest variability of subcortical regions and substructures of the amygdala and hippocampus.

In all subcortical regions ICCs were ≥0.95 and bias <2%. In all hippocampal subfields and nuclei of the amygdala included in analysis ICCs were ≥0.88, except the right medial nucleus with an ICC = 0.76. Bias was within 5.6%. The lowest ICC for assessment of cortical thickness was ICC = 0.80 in the right entorhinal cortex, the highest bias was 2.2% in the left entorhinal cortex. Supplementary Table 1 available at https://doi.org/10.1192/bjp.2018.224 provides test–retest data for all regions analysed.

### Effects of ECT on subcortical regions, nuclei of the right amygdala, and subfields of the right hippocampus

**Fig. 1:**
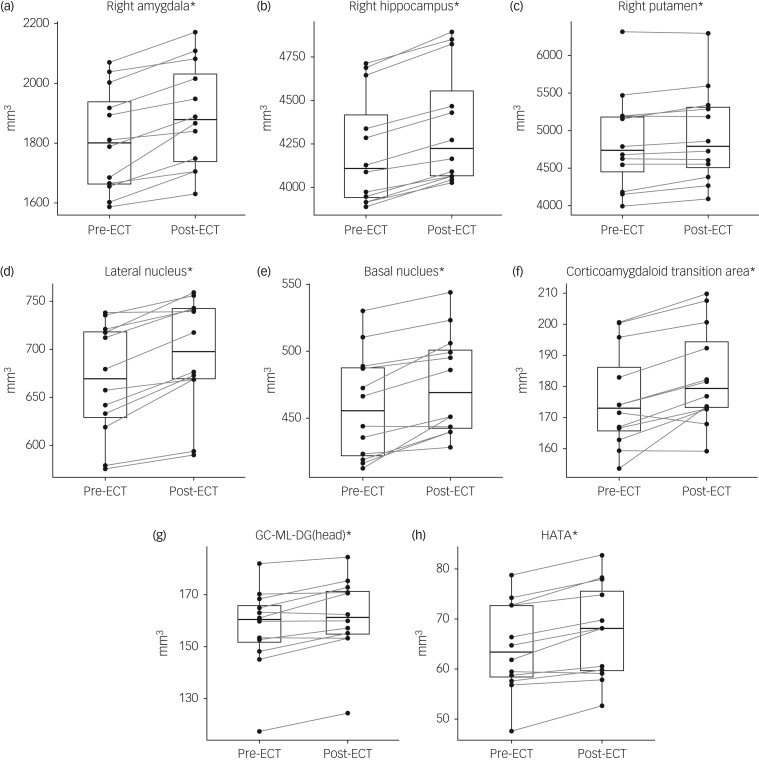
Changes in the volume of subcortical regions (a–c), nuclei of the right amygdala (d–f) and subfields of the right hippocampus (g, h) after electroconvulsive therapy (ECT).

Repeated-measures ANOVA indicated an interaction of time and region (*F*(6,66) = 11.60, *P* < 0.0001) and a main effect of time (*F*(1,11) = 37.40, *P* < 0.0001) with higher volumes post-ECT compared with baseline (*P* < 0.0001) in subcortical regions of the right hemisphere (Table 2, Fig. 1(a–c)). Testing of individual subcortical regions revealed higher volumes after ECT compared with baseline scans in the right hippocampus by 3.3% (s.d. =  0.7%) (*t*(11) = 13.83, *P*_FWE_ < 0.0001), the right amygdala by 4.6% (s.d. = 2.6%) (*t*(11) = 6.50, *P*_FWE_ < 0.001) and the right putamen by 1.6% (s.d. =  1.7%) (*t*(11) = 3.33, *P*_FWE_ = 0.05). No significant effects of ECT were found in subcortical regions of the left hemisphere.

(a) Right amygdala; (b) right hippocampus; (c) right putamen; (d) right lateral nucleus; (e) right basal nucleus; (f) right corticoamygdaloid transition area; (g) granule cell and molecular layer of the dentate gyrus in the right hippocampal head (GC-ML-DG(head)); and (h) right hippocampal–amygdaloid transition area (HATA). *Results for a–h were all significant (*P*_FWE_ ≤ 0.05 (Bonferroni corrected)). Each dot represents the volume of the respective brain region in an individual patient.

**Table 2:** Changes in volumes of subcortical regions, nuclei of the amygdala and hippocampal subfields in the right hemisphere after electroconvulsive therapy (ECT) treatment^a^

	Mean (s.d.)			
	Pre-ECT MRI 1 and 2, mm^3^	Post-ECT MRI 3, mm^3^	Change, mm^3^	Change, %
Subcortical regions				
Right accumbens area	610.7 (125.6)	619.9 (134.5)	9.1 (21.6)	1.3 (3.5)
Right amygdala*	1810.0 (173.2)	1892.2 (176.9)	82.3 (43.9)	4.6 (2.6)
Right caudate	3573.4 (396.5)	3617.7 (422.0)	44.4 (69.5)	1.2 (1.9)
Right hippocampus*	4209.4 (319.0)	4348.8 (337.2)	139.4 (34.9)	3.3 (0.7)
Right pallidum^†^	1905.8 (180.7)	1876.8 (178.0)	−29.0 (34.3)	−1.5 (1.9)
Right putamen*	4858.3 (656.1)	4932.2 (636.3)	73.9 (77.0)	1.6 (1.7)
Right thalamus^†^	7368.4 (814.1)	7475.5 (746.9)	107.1 (116.0)	1.6 (1.8)
Nuclei of the right amygdala				
Accessory basal nucleus	290.0 (31.8)	299.0 (31.2)	9.0 (14.0)	3.3 (5.2)
Anterior amygdaloid area	57.6 (8.0)	59.8 (8.6)	2.2 (3.5)	3.9 (6.0)
Basal nucleus*	458.8 (39.5)	475.6 (38.0)	16.7 (11.3)	3.7 (2.7)
Central nucleus	44.8 (8.4)	45.0 (7.1)	0.3 (5.3)	1.9 (15.1)
Cortical nucleus	28.0 (3.5)	28.2 (3.9)	0.2 (1.9)	0.7 (7.1)
Corticoamygdaloid transition area*	175.8 (15.9)	183.2 (16.0)	7.4 (5.8)	4.3 (3.7)
Lateral nucleus*	667.5 (58.4)	694.1 (58.8)	26.6 (15.0)	4.0 (2.3)
Medial nucleus	22.5 (5.8)	22.1 (5.3)	−0.4 (3.6)	−0.4 (17.2)
Paralaminar nucleus^†^	45.6 (4.7)	47.2 (5.0)	1.6 (1.5)	3.6 (3.7)
Subfields of the right hippocampus				
Cornu ammonis 1 body	131.0 (23.6)	128.8 (25.0)	−2.2 (4.3)	−1.8 (3.2)
Cornu ammonis 1 head	548.6 (34.4)	551.2 (35.9)	2.6 (9.1)	0.5 (1.6)
Cornu ammonis 3 body	116.5 (34.8)	113.6 (31.6)	−2.9 (4.3)	−1.8 (3.8)
Cornu ammonis 3 head	134.7 (23.4)	135.2 (21.3)	0.5 (4.4)	0.7 (3.4)
Cornu ammonis 4 body	125.0 (15.5)	125.1 (17.0)	0.1 (5.9)	0.1 (4.5)
Cornu ammonis 4 head^†^	133.4 (14.8)	137 (13.9)	3.6 (3.9)	2.8 (3.1)
Hippocampal fimbria	55.2 (12.2)	56.3 (12.7)	1.0 (5.5)	2.3 (12.2)
Granule cell and molecular layer of the dentate gyrus (body)	134 (15.8)	134.3 (19.1)	0.4 (8.2)	0.2 (5.6)
Granule cell and molecular layer of the dentate gyrus (head)*	157.4 (16.1)	161.8 (15.3)	4.5 (3.8)	3.0 (2.6)
Hippocampal–amygdaloid transition area*	64.3 (9.0)	67.5 (9.6)	3.1 (1.9)	4.9 (3.2)
Hippocampal fissure	161.2 (32.0)	150.8 (24.7)	−10.4 (13.3)	−5.5 (8.2)
Hippocampal tail	571.1 (49.3	566.5 (50.9	−4.6 (14.5	−0.8 (2.6
Molecular layer (body)	225.6 (30.5)	223.9 (32.5)	−1.7 (8.2)	−0.8 (3.6)
Molecular layer (head)^†^	337.9 (25.3)	342.2 (26.9)	4.2 (4.8)	1.2 (1.4)
Parasubiculum	61.0 (15.4)	60.3 (15.0)	−0.7 (2.4)	−1.0 (4.5)
Presubiculum (body)	135.2 (27.4)	133.4 (28.7)	−1.8 (7.6)	−1.4 (5.7)
Presubiculum (head)	133.3 (18.2)	132.9 (17.8)	−0.4 (5.0)	−0.2 (3.8)
Subiculum (body)	214.0 (29.2)	215.5 (29.8)	1.5 (7.7)	0.7 (3.7)
Subiculum (head)	184.2 (24.9)	184.5 (22.8)	0.3 (5.7)	0.3 (3.4)

MRI, magnetic resonance imaging.

a. Significant increases in several subcortical regions, subfields and nuclei of the right hippocampus and amygdala were observed between baseline and post-ECT scans. Most other regions also showed an increase in volume, which was not significant after Bonferroni correction for testing multiple regions. Pre-ECT data were calculated as the average of both baseline scans. Only data from participants with available post-ECT MRI data are shown (*n* = 12).

* Significant at *P*_FWE_ ≤ 0.05 (Bonferroni corrected); † FDR controlled at q*≤0.05 (Benjamini–Hochberg procedure).

Based on these results, repeated-measures ANOVA was performed for nuclei of the right amygdala and subfields of the right hippocampus. An interaction of region and time (*F*(27,297) = 9.98, *P* < 0.0001) and a main effect of time (*F*(1,11) = 11.56, *P* = 0.006) were observed. Analysis of individual nuclei of the amygdala showed increased volumes in the lateral nucleus by 4.0% (s.d. =  2.3%) (*t*(11) = 6.16, *P*_FWE_ = 0.002), basal nucleus by 3.7% (s.d. =  2.7%) (*t*(11) = 5.13, *P*_FWE_ < 0.01) and corticoamygdaloid transition area (CTA) by 4.3% (s.d. =  3.7%) (*t*(11) = 4.41, *P*_FWE_ = 0.03) (Table 2, Fig. 1(d–f)). Among the subfields of the right hippocampus, increased volumes following ECT were observed in the hippocampal–amygdaloid transition area (HATA) by 4.9% (s.d. = 3.2%) (*t*(11) = 5.72, *P*_FWE_ < 0.004) and the granule cell and molecular layer of the dentate gyrus in the hippocampal head by 3.0% (s.d. =  2.6%) (*t*(11) = 4.11, *P*_FWE_ < 0.05) (Table 2, Fig. 1(g, h)).

**Table 3:** Changes in cortical thickness of the right hemisphere after electroconvulsive therapy (ECT)^a^

Cortical thickness	Mean (s.d.)			
	Pre-ECT MRI 1 and 2, μm	Post-ECT MRI 3, μm	Change, μm	Change, %
Right banks of the superior temporal sulcus*	2519 (162)	2598 (148)	79 (61)	3.2 (2.5)
Right caudal anterior cingulate cortex^†^	2710 (205)	2769 (220)	59 (65)	2.2 (2.4)
Right fusiform gyrus*	2714 (125)	2770 (126)	56 (49)	2.1 (1.9)
Right inferior parietal cortex*	2580 (97)	2636 (111)	56 (34)	2.2 (1.3)
Right inferior temporal gyrus*	2769 (94)	2831 (100)	62 (43)	2.2 (1.5)
Right insula*	3111 (201)	3228 (221)	117 (53)	3.8 (1.7)
Right lateral occipital cortex^†^	2253 (159)	2287 (158)	34 (44)	1.5 (2)
Right medial orbitofrontal cortex^†^	2467 (129)	2509 (114)	42 (43)	1.8 (1.8)
Right middle temporal gyrus^†^	2867 (154)	2931 (172)	64 (59)	2.2 (2.0)
Right parahippocampal gyrus^†^	2781 (189)	2820 (212)	39 (47)	1.4 (1.7)
Right pars opercularis^†^	2616 (142)	2663 (153)	47 (47)	1.8 (1.8)
Right pars triangularis^†^	2456 (167)	2497 (168)	41 (42)	1.7 (1.8)
Right posterior cingulate cortex^†^	2578 (128)	2606 (132)	28 (36)	1.1 (1.4)
Right postcentral gyrus*	2110 (104)	2151 (120)	41 (34)	1.9 (1.6)
Right precuneus^†^	2505 (122)	2554 (134)	49 (43)	1.9 (1.7)
Right superior frontal gyrus^†^	2858 (140)	2893 (133)	35 (40)	1.2 (1.4)
Right superior parietal cortex^†^	2286 (98)	2334 (89)	48 (50)	2.1 (2.3)
Right superior temporal gyrus*	2842 (127)	2931 (127)	89 (59)	3.2 (2.1)
Right supramarginal gyrus*	2546 (117)	2611 (125)	65 (35)	2.5 (1.4)
Right temporal pole*	4005 (337)	4116 (315)	111 (87)	2.9 (2.4)

MRI, magnetic resonance imaging.

a. Regions with significant or trend-level increases in cortical thickness are tabulated here. Data on the remaining cortical regions can be found in Supplementary Table 1. Pre-ECT data were calculated as the average of both baseline scans. Only data from participants with available post-ECT MRI data are shown (*n* = 13).

* Significant at *P*_FWE_≤0.05 (Bonferroni corrected); † FDR controlled at q*≤0.05 (Benjamini–Hochberg procedure).

### Effects of ECT on cortical thickness

**Fig. 2:**
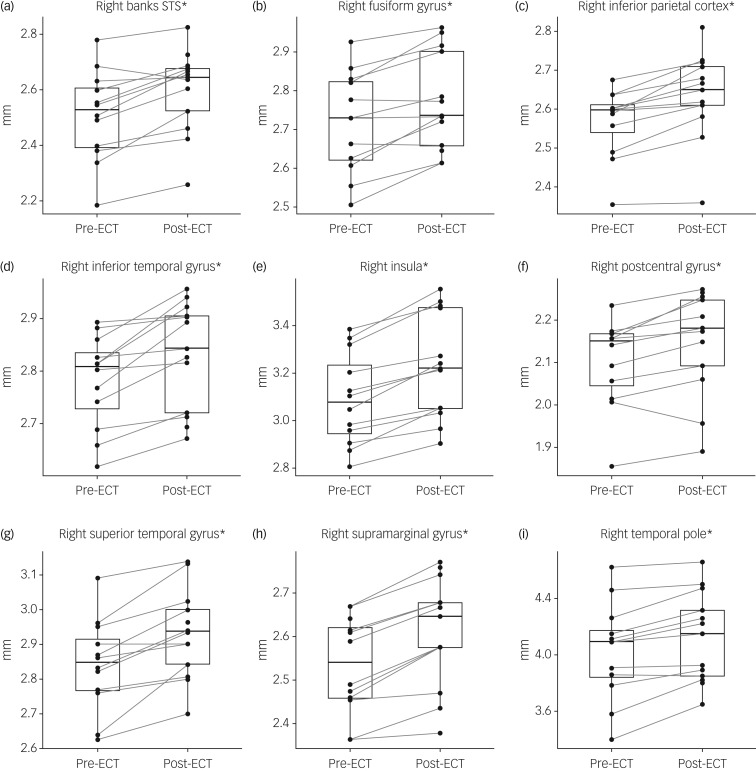
Changes in cortical thickness after electroconvulsive therapy (ECT).

There was an interaction of time and region (*F*(33,396) = 2.67, *P* < 0.0001) and a main effect of time (*F*(1,12) = 51.91, *P* < 0.0001) in cortical thickness data of the right hemisphere. In the left hemisphere a small effect of time could be observed (*F*(1,12) = 5.58, *P* = 0.04), but no significant interaction of time and region. In *post hoc* tests an increase in cortical thickness after ECT could be seen in the following regions of the right hemisphere: insula (*t*(12) = 7.95, *P*_FWE_ = 0.0001), supramarginal gyrus (*t*(12) = 6.68, *P*_FWE_ < 0.001), inferior parietal cortex (*t*(12) = 6.03, *P*_FWE_ = 0.002), superior temporal gyrus (*t*(12) = 5.47, *P*_FWE_ < 0.005), inferior temporal gyrus (*t*(12) = 5.18, *P*_FWE_ < 0.01), banks of the superior temporal sulcus (*t*(12) = 4.65, *P*_FWE_ = 0.02), temporal pole (*t*(12) = 4.61, *P*_FWE_ = 0.02), postcentral gyrus (*t*(12) = 4.27, *P*_FWE_ = 0.04) and fusiform gyrus (*t*(12) = 4.12, *P*_FWE_ < 0.05) (Fig. 2(a–i)).

(a) Right bank superior temporal sulcus (STS); (b) right fusiform gyrus; (c) right inferior parietal cortex; (d) right inferior temporal gyrus; (e) right insula; (f) right postcentral gyrus; (g) right superior temporal gyrus; (h) right supramarginal gyrus; and (i) right temporal pole. *Results for a–i were all significant (*P*_FWE_ ≤ 0.05 (Bonferroni corrected)). Each dot represents the thickness of the respective brain region in an individual patient.

An increase in cortical thickness after ECT was observed in 66 out of 68 cortical regions with the strongest effect in the right insula, where cortical thickness increased by 3.8% (s.d. = 1.7%). Data on all cortical areas can be found in supplementary Table 2.

### Correlation of imaging data with change in depressive symptoms

Negative correlations between pre-ECT volumes and symptom improvement were found for the right thalamus (*r* = −0.79, *P* = 0.001) and right nucleus accumbens (*r* = −0.72, *P* = 0.004) among subcortical regions and for the following hippocampal subfields: right presubiculum (*r* = −0.72, *P* = 0.004), left presubiculum (*r* = −0.70, *P* = 0.006) and right subiculum body (*r* = −0.68, *P* = 0.007) and head (*r* = −0.63, *P* = 0.016). A positive correlation between change in volume and symptom improvement was found for the left subiculum head (*r* = 0.90, *P* < 0.0001) and right parasubiculum (*r* = 0.74, *P* = 0.006). No correlations were found between cortical thickness and clinical response.

## Discussion

In the current study the effect of increased volume after ECT in the hippocampus, amygdala, putamen, as well as cortical thickness in several areas could be replicated. All imaging outcomes acquired had test–retest reliability ranging from high to excellent (supplementary Table 1). The allocation of structural changes to specific subfields of the hippocampus and nuclei of the amygdala, and the comprehensive analysis of changes in cortical thickness are novel and are discussed in further detail. In line with a mega-analysis of ECT effects on hippocampal volumes,^2^ there was a lateralisation of structural changes, possibly because of right unilateral placement of electrodes during stimulation.

### Effects of ECT on subfields of the right hippocampus

Following ECT, increases in the volume of the HATA and the granule cell and molecular layer of the dentate gyrus of the hippocampal head were found. Trend-level effects were observed in the cornu ammonis 4 and molecular layer of the hippocampal head. These findings are in agreement with recently reported changes in bilateral cornu ammonis 4 and granule cell layer following bitemporal ECT^9^ and suggest that the effect may be sensitive to electrode placement.

In animal models chronic corticosterone treatment reduces neurogenesis in the dentate gyrus that can be reversed by ECT.^19^ On the other hand, inhibition of neurogenesis in this region interferes with endocrinological recovery from stress and induces a depressive phenotype.^20^ Next to effects on neurogenesis the structural changes following electroconvulsive seizures could be attributed to increases in dendritic spine and synapse densities, as shown in the adult hippocampus of rats.^21^ In unmedicated major depression a reduced volume and number of granule neurons in the dentate gyrus is observed post-mortem, which is not found in patients treated with selective serotonin reuptake inhibitors.^22^ In line with observed changes of serotonin 1a receptors during ECT in patients with depression,^23^ the expression of this receptor in the dentate gyrus is essential for the behavioural and hypothalamic–pituitary–adrenal axis response to antidepressants.^24^ Altogether, this evidence highlights the association of phenotypical alterations in these hippocampal subfields with stressful experience and mental illness that may be plastically modified by antidepressant treatment in general and ECT specifically.

### Effects of ECT on nuclei of the right amygdala

Significant increases in the volumes of the basal, and lateral nuclei, as well as the CTA were observed after ECT. The current results were obtained using newly implemented segmentation of amygdala nuclei in FreeSurfer.^5^ One previous study applied shape analysis and reported surface deformations in the basolateral aspect of the left amygdala and the dorsomedial aspect of the amygdala bilaterally in both hemispheres following right unilateral ECT.^4^ Comparison with current results is impaired by missing information on the effects on volume, such that reported surface deformations may reflect directions with room for expansion rather than localised increases in volume. Similar to the dentate gyrus, recent evidence points to the presence of adult neurogenesis in the basolateral amygdala, a region that has been classically implicated in Pavlovian fear conditioning.^25^ Induction of cyclic adenosine monophosphate response element-binding protein in the basolateral amygdala resulted in an antidepressant behavioural response.^26^ Animal models of depression demonstrated increased expression of voltage-gated calcium channels in the basolateral amygdala and dentate gyrus, which was decreased following ECT.^27^ Interestingly, serotonin was found to reduce excitatory neurotransmission and calcium influx in the basolateral amygdala via serotonin 1a receptors,^28^ and reductions of serotonin transporters as a surrogate of serotonergic transmission in amygdala of patients with depression have been found.^29^

### Effects of ECT on cortical thickness

A widespread increase in cortical thickness of the right hemisphere was observed. The lateralisation of the effect on cortical thickness to the right hemisphere is in line with reported grey matter volume changes in a mixed sample of patients treated with unilateral and bilateral stimulation.^3^ Correspondingly, asymmetry of changes in temporal cortex was less pronounced in a sample of patients treated with bilateral ECT only.^14^ One region for which no significant changes were reported in previous studies despite its well-established role in major depression is the precuneus. Our findings are in line with a recent study that found grey matter volume in the precuneus of predictive value for ECT response in patients with depression.^30^ Furthermore, default mode network coherence was reported to be reduced in the precuneus of patients with depression, which was reversed after ECT in individuals who were responders.^31^ The increase of cortical thickness in the precuneus may be associated with the reversal of alterations in major depression and deserves further investigation.

### Correlation of brain structure and clinical improvement

As can be seen in Figs 1 and 2 brain regions with significant changes after ECT seem to increase to a similar extent in most patients. This implies an effect of ECT on neuroplasticity that is consistent across patients and independent of baseline values, which makes correlations of changes in these regions with antidepressant response unlikely. Accordingly, correlation of changes in the volume of hippocampal subfields and clinical improvement were not found by Cao *et al*.^9^ In the exploratory analysis of our sample, such correlations could be seen in the left subiculum head and right parasubiculum, regions that did not show significant increases in volume after ECT. Despite the strong effect seen for the left subiculum head, replication is needed, especially since Cao *et al* did not find any effect for the whole subiculum. On the other hand, correlating baseline differences in volumes with antidepressant response promises to influence clinical practice if it allows for predicting which patients will benefit from ECT. Here, we found negative correlations for the right thalamus and nucleus accumbens, which needs replication before speculating about potential implications. Of note, we could replicate the negative correlation of baseline volume of the right subiculum and presubiculum with antidepressant response. As this region, at the interface between the hippocampus and cortex, does not increase in volume after ECT, its potential as a trait marker for ECT response warrants further exploration.

### Limitations

An advantage of the design is that the participants studied were highly representative of patients receiving ECT for treatment of depression in clinical routine. Recruitment of suitable patients was difficult and only a modest number of patients could be included, which is seen in many imaging studies on ECT. Therefore, cross-sectional comparisons between participants were omitted for reasons of power (for example those who respond versus those who do not respond). However, the likelihood of false positive error is very low given the conservative statistical correction for multiple comparisons applied and the high agreement of results with the literature.

A limitation of the present findings is the pre–post study design lacking a control group. Therefore, the differentiation of effects as a result of time and the in-patient setting is not possible. Another issue is the inclusion of patients with ongoing psychopharmacological treatment. These limitations were imposed by ethical considerations that make the discontinuation of medication and delay of active treatment difficult to justify. These limitations were partly compensated by requiring stable medication for 10 days prior to inclusion and performing two MRI scans at baseline, which underpin the stability of outcome measures. Owing to the multiple scans performed and expertise in processing data, deviations in individual scans could be easily identified and were excluded to avoid bias arising from the introduction of manual corrections of structural data. Therefore, the application of structural imaging to research and clinical practice is limited by the requirement of accurate segmentation of brain tissue. Finally, the neuroplastic changes after ECT measured with the presently available structural MRI method do not allow for a differentiation of the underlying cellular mechanisms, such that it remains to be determined to what extent they can be attributed to neurogenesis, synaptogenesis, gliogenesis, morphological changes or changes in vascular tissues.

### Implications

Following right unilateral ECT, increases of the volume of the right amygdala and hippocampus, as well as of the thickness of the right insula, temporal and parietal cortex were observed. The increase in volume was localised to the basolateral amygdala, and CTA, as well as the HATA, and the granule cell and molecular layer in the dentate gyrus of the hippocampal head. These subregions are associated with alterations found in depression and following stressful experience and retain a high potential for neuroplasticity in adulthood. The observed structural effects support a modification of these phenotypes by ECT and contribute to our understanding of its mechanism of action.

## Supplementary material

For supplementary material accompanying this paper visit https://doi.org/10.1192/bjp.2018.224. click here to view supplementary material
