# Supplementary material for: Structural changes in amygdala nuclei, hippocampal subfields and cortical thickness following electroconvulsive therapy in treatment-resistant depression: longitudinal analysis
Source: Br J Psychiatry. 2019 Mar;214(3):159–67. doi: 10.1192/bjp.2018.224 (PMC6383756; doi:10.1192/bjp.2018.224)
Supplement: Supplementary file 1 [file S0007125018002246sup001.docx]

| **Subcortial regions** | **Mean change (%)** | **Variability (%)** | **ICC** |
| --- | --- | --- | --- |
| Right accumbens area | 0.39 | 3.69 | 0.99 |
| Right amygdala | -0.30 | 2.17 | 0.97 |
| Right caudate | 0.61 | 1.62 | 0.99 |
| Right hippocampus | 0.35 | 0.99 | 0.99 |
| Right pallidum | 0.76 | 2.75 | 0.96 |
| Right putamen | 0.27 | 1.12 | 1.00 |
| Right thalamus | 0.37 | 1.07 | 1.00 |
| Left accumbens area | -1.89 | 10.70 | 0.98 |
| Left amygdala | -1.63 | 2.83 | 0.95 |
| Left caudate | 0.39 | 1.20 | 0.99 |
| Left hippocampus | -0.01 | 1.56 | 0.98 |
| Left pallidum | 0.52 | 2.13 | 0.99 |
| Left putamen | 0.75 | 1.55 | 0.99 |
| Left thalamus | 0.76 | 1.42 | 0.99 |
| **Nuclei of the right amygdala** | **Mean change (%)** | **Variability (%)** | **ICC** |
| Accessory basal nucleus | -1.64 | 3.47 | 0.95 |
| Anterior amygdaloid area | -2.1 | 6.72 | 0.9 |
| Basal nucleus | -1.05 | 3.99 | 0.9 |
| Central nucleus | -2.89 | 7.82 | 0.88 |
| Cortical nucleus | -0.72 | 5.17 | 0.91 |
| Corticoamygdaloid transition area | -1.22 | 2.34 | 0.95 |
| Lateral nucleus | -1.86 | 2.46 | 0.93 |
| Medial nucleus | -3.53 | 15.52 | 0.76 |
| Paralaminar nucleus | -0.45 | 2.72 | 0.95 |
| **Subfields of the right hippocampus** | **Mean change (%)** | **Variability (%)** | **ICC** |
| CA1 body | 1.03 | 1.92 | 0.99 |
| CA1 head | 0.05 | 1.62 | 0.98 |
| CA3 body | -0.36 | 3.43 | 0.99 |
| CA3 head | -0.18 | 2.66 | 0.99 |
| CA4 body | -0.64 | 3.37 | 0.94 |
| CA4 head | -0.74 | 1.94 | 0.98 |
| Hippocampal fimbria | -5.58 | 12.24 | 0.92 |
| GC-ML-DG (body) | -0.61 | 3.24 | 0.93 |
| GC-ML-DG (head) | -0.34 | 2.34 | 0.98 |
| Hippocampal-amygdaloid transition area | -2.95 | 5.48 | 0.89 |
| Hippocampal fissure | -0.97 | 7.19 | 0.94 |
| Hippocampal tail | -0.44 | 3.82 | 0.92 |
| Molecular layer (body) | -0.02 | 2.07 | 0.97 |
| Molecular layer (head) | 0.27 | 1.6 | 0.98 |
| Parasubiculum | 2.86 | 3.77 | 0.98 |
| Presubiculum (body) | 0.96 | 3.76 | 0.98 |
| Presubiculum (head) | 1.94 | 4.09 | 0.96 |
| Subiculum (body) | -0.32 | 4.06 | 0.95 |
| Subiculum (head) | 0.48 | 2.46 | 0.98 |
| **Cortical area** | **Mean change (%)** | **Variability (%)** | **ICC** |
| Right banks of the superior temporal sulcus | 0.12 | 0.02 | 0.96 |
| Right caudal anterior cingulate cortex | 0.44 | 0.02 | 0.98 |
| Right caudal middle frontal gyrus | 0.10 | 0.01 | 0.96 |
| Right cuneus | 0.31 | 0.03 | 0.89 |
| Right entorhinal cortex | -0.29 | 0.07 | 0.80 |
| Right frontal pole | 0.57 | 0.05 | 0.85 |
| Right fusiform gyrus | -0.85 | 0.03 | 0.93 |
| Right inferior parietal cortex | -0.35 | 0.02 | 0.92 |
| Right inferior temporal gyrus | 0.29 | 0.01 | 0.96 |
| Right insula | 0.54 | 0.02 | 0.96 |
| Right isthmus cingulate cortex | 0.57 | 0.01 | 0.98 |
| Right lateral occipital cortex | 0.52 | 0.02 | 0.97 |
| Right lateral orbitofrontal cortex | 0.56 | 0.01 | 0.96 |
| Right lingual gyrus | -0.55 | 0.03 | 0.96 |
| Right medial orbitofrontal cortex | 0.37 | 0.01 | 0.97 |
| Right middle temporal gyrus | 0.43 | 0.02 | 0.97 |
| Right parahippocampal gyrus | -1.14 | 0.03 | 0.84 |
| Right paracentral lobule | -0.33 | 0.02 | 0.92 |
| Right pars opercularis | -0.10 | 0.02 | 0.94 |
| Right pars orbitalis | -0.13 | 0.03 | 0.90 |
| Right pars triangularis | 0.27 | 0.02 | 0.96 |
| Right pericalcarine cortex | -0.24 | 0.06 | 0.88 |
| Right postcentral gyrus | -0.38 | 0.03 | 0.88 |
| Right posterior cingulate cortex | 0.33 | 0.02 | 0.94 |
| Right precentral gyrus | -0.65 | 0.03 | 0.86 |
| Right precuneus | -0.15 | 0.02 | 0.90 |
| Right rostral anterior cingulate cortex | -0.77 | 0.02 | 0.95 |
| Right rostral middle frontal gyrus | 0.73 | 0.02 | 0.96 |
| Right superior frontal gyrus | 0.18 | 0.01 | 0.98 |
| Right superior parietal cortex | 0.43 | 0.03 | 0.83 |
| Right superior temporal gyrus | -0.23 | 0.01 | 0.96 |
| Right supramarginal gyrus | 0.28 | 0.02 | 0.92 |
| Right temporal pole | -0.90 | 0.02 | 0.97 |
| Right transverse temporal cortex | 0.73 | 0.04 | 0.92 |
| Left banks of the superior temporal sulcus | -0.14 | 0.01 | 0.97 |
| Left caudal anterior cingulate cortex | -1.14 | 0.02 | 0.92 |
| Left caudal middle frontal gyrus | 0.03 | 0.02 | 0.92 |
| Left cuneus | 1.12 | 0.02 | 0.96 |
| Left entorhinal cortex | 2.17 | 0.03 | 0.88 |
| Left frontal pole | -1.23 | 0.02 | 0.88 |
| Left fusiform gyrus | -0.80 | 0.02 | 0.91 |
| Left inferior parietal cortex | -0.45 | 0.02 | 0.92 |
| Left inferior temporal gyrus | 0.27 | 0.01 | 0.98 |
| Left insula | 0.09 | 0.01 | 0.97 |
| Left isthmus cingulate cortex | 0.18 | 0.03 | 0.95 |
| Left lateral occipital cortex | 0.22 | 0.02 | 0.92 |
| Left lateral orbitofrontal cortex | -0.05 | 0.02 | 0.96 |
| Left lingual gyrus | -0.28 | 0.04 | 0.85 |
| Left medial orbitofrontal cortex | 0.52 | 0.01 | 0.95 |
| Left middle temporal gyrus | -0.06 | 0.01 | 0.95 |
| Left parahippocampal gyrus | -0.36 | 0.03 | 0.94 |
| Left paracentral lobule | -0.77 | 0.02 | 0.99 |
| Left pars opercularis | 0.24 | 0.01 | 0.97 |
| Left pars orbitalis | -0.30 | 0.02 | 0.98 |
| Left pars triangularis | 0.76 | 0.02 | 0.92 |
| Left pericalcarine cortex | 0.76 | 0.04 | 0.90 |
| Left postcentral gyrus | 0.33 | 0.02 | 0.96 |
| Left posterior cingulate cortex | 0.77 | 0.02 | 0.91 |
| Left precentral gyrus | 0.19 | 0.03 | 0.87 |
| Left precuneus | -0.34 | 0.02 | 0.90 |
| Left rostral anterior cingulate cortex | 1.05 | 0.02 | 0.95 |
| Left rostral middle frontal gyrus | 0.37 | 0.01 | 0.97 |
| Left superior frontal gyrus | -0.13 | 0.01 | 0.97 |
| Left superior parietal cortex | 0.23 | 0.03 | 0.84 |
| Left superior temporal gyrus | -0.11 | 0.01 | 0.97 |
| Left supramarginal gyrus | 0.33 | 0.01 | 0.93 |
| Left temporal pole | 0.61 | 0.03 | 0.92 |
| Left transverse temporal cortex | 1.23 | 0.04 | 0.88 |

**Supplementary Table 1. Test-retest reliability of imaging outcome measures.** Data obtained from the two MRI scans obtained before ECT was analyzed (n=12 for cortical thickness, n=11 for subcortical regions, nuclei of the right amygdala, and subfields of the right hippocampus). Mean change and variability were calculated as the mean and standard deviation of the relative difference between the two scans. ICC, intraclass correlation coefficient.

| **Cortical area** | **Pre-ECT MRI 1 & 2 (µm)** | **Post-ECT MRI 3 (µm)** | **Change (µm)** | **Change (%)** |
| --- | --- | --- | --- | --- |
| Right banks of the superior temporal sulcus* | 2519±162 | 2598±148 | 79±61 | 3.2±2.5 |
| Right caudal anterior cingulate cortex† | 2710±205 | 2769±220 | 59±65 | 2.2±2.4 |
| Right caudal middle frontal gyrus | 2669±128 | 2702±132 | 33±50 | 1.2±1.9 |
| Right cuneus | 1872±138 | 1909±157 | 37±58 | 1.9±3.1 |
| Right entorhinal cortex | 3535±318 | 3626±279 | 91±139 | 2.7±4.3 |
| Right frontal pole | 2699±233 | 2733±224 | 33±87 | 1.3±3.3 |
| Right fusiform gyrus* | 2714±125 | 2770±126 | 56±49 | 2.1±1.9 |
| Right inferior parietal cortex* | 2580±97 | 2636±111 | 56±34 | 2.2±1.3 |
| Right inferior temporal gyrus* | 2769±94 | 2831±100 | 62±43 | 2.2±1.5 |
| Right insula* | 3111±201 | 3228±221 | 117±53 | 3.8±1.7 |
| Right isthmus cingulate cortex | 2412±209 | 2427±208 | 15±40 | 0.6±1.7 |
| Right lateral occipital cortex† | 2253±159 | 2287±158 | 34±44 | 1.5±2 |
| Right lateral orbitofrontal cortex | 2594±136 | 2620±135 | 25±42 | 1±1.6 |
| Right lingual gyrus | 2004±164 | 2041±148 | 37±55 | 2±2.8 |
| Right medial orbitofrontal cortex† | 2467±129 | 2509±114 | 42±43 | 1.8±1.8 |
| Right middle temporal gyrus† | 2867±154 | 2931±172 | 64±59 | 2.2±2 |
| Right parahippocampal gyrus† | 2781±189 | 2820±212 | 39±47 | 1.4±1.7 |
| Right paracentral lobule | 2617±129 | 2648±143 | 32±59 | 1.2±2.3 |
| Right pars opercularis† | 2616±142 | 2663±153 | 47±47 | 1.8±1.8 |
| Right pars orbitalis | 2773±149 | 2785±167 | 13±60 | 0.4±2.2 |
| Right pars triangularis† | 2456±167 | 2497±168 | 41±42 | 1.7±1.8 |
| Right pericalcarine cortex | 1545±151 | 1573±147 | 28±77 | 2±5.1 |
| Right postcentral gyrus* | 2110±104 | 2151±120 | 41±34 | 1.9±1.6 |
| Right posterior cingulate cortex† | 2578±128 | 2606±132 | 28±36 | 1.1±1.4 |
| Right precentral gyrus | 2563±130 | 2589±134 | 26±59 | 1±2.3 |
| Right precuneus† | 2505±122 | 2554±134 | 49±43 | 1.9±1.7 |
| Right rostral anterior cingulate cortex | 3033±178 | 3078±209 | 45±88 | 1.5±2.7 |
| Right rostral middle frontal gyrus | 2414±113 | 2435±117 | 22±44 | 0.9±1.8 |
| Right superior frontal gyrus† | 2858±140 | 2893±133 | 35±40 | 1.2±1.4 |
| Right superior parietal cortex† | 2286±98 | 2334±89 | 48±50 | 2.1±2.3 |
| Right superior temporal gyrus* | 2842±127 | 2931±127 | 89±59 | 3.2±2.1 |
| Right supramarginal gyrus | 2546±117 | 2611±125 | 65±35 | 2.5±1.4 |
| Right temporal pole* | 4005±337 | 4116±315 | 111±87 | 2.9±2.4 |
| Right transverse temporal cortex | 2327±214 | 2349±207 | 22±86 | 1±3.5 |
| Left banks of the superior temporal sulcus | 2491±119 | 2524±117 | 33±68 | 1.4±2.9 |
| Left caudal anterior cingulate cortex | 2880±188 | 2951±245 | 71±70 | 2.4±2.4 |
| Left caudal middle frontal gyrus | 2675±116 | 2686±118 | 10±67 | 0.4±2.6 |
| Left cuneus | 1799±144 | 1827±148 | 28±45 | 1.6±2.5 |
| Left entorhinal cortex | 3464±218 | 3424±196 | -41±145 | -1.1±4.1 |
| Left frontal pole | 2790±129 | 2799±127 | 8±87 | 0.3±3.1 |
| Left fusiform gyrus | 2773±129 | 2793±157 | 20±38 | 0.7±1.4 |
| Left inferior parietal cortex | 2536±118 | 2561±121 | 25±54 | 1±2.2 |
| Left inferior temporal gyrus | 2853±140 | 2860±155 | 7±48 | 0.2±1.7 |
| Left insula | 3080±153 | 3092±168 | 12±64 | 0.4±2.1 |
| Left isthmus cingulate cortex | 2384±232 | 2386±215 | 3±39 | 0.2±1.6 |
| Left lateral occipital cortex | 2224±129 | 2237±147 | 13±47 | 0.5±2.1 |
| Left lateral orbitofrontal cortex | 2671±151 | 2662±151 | -8±65 | -0.3±2.4 |
| Left lingual gyrus | 1955±113 | 1995±107 | 40±52 | 2.1±2.7 |
| Left medial orbitofrontal cortex | 2391±112 | 2433±123 | 42±84 | 1.8±3.5 |
| Left middle temporal gyrus | 2853±100 | 2858±102 | 4±59 | 0.2±2.1 |
| Left parahippocampal gyrus | 2810±276 | 2822±296 | 13±74 | 0.4±2.7 |
| Left paracentral lobule | 2518±189 | 2527±215 | 9±76 | 0.3±3.1 |
| Left pars opercularis | 2634±146 | 2656±146 | 22±52 | 0.9±2 |
| Left pars orbitalis | 2764±227 | 2774±247 | 11±66 | 0.4±2.4 |
| Left pars triangularis | 2439±141 | 2452±141 | 13±46 | 0.6±1.9 |
| Left pericalcarine cortex | 1555±143 | 1554±151 | -1±65 | 0±4.3 |
| Left postcentral gyrus | 2143±115 | 2176±107 | 33±38 | 1.6±1.8 |
| Left posterior cingulate cortex | 2581±145 | 2612±144 | 31±36 | 1.2±1.4 |
| Left precentral gyrus | 2656±126 | 2664±108 | 8±50 | 0.3±1.9 |
| Left precuneus | 2511±116 | 2548±132 | 37±44 | 1.5±1.8 |
| Left rostral anterior cingulate cortex | 2802±151 | 2820±173 | 18±61 | 0.6±2.1 |
| Left rostral middle frontal gyrus | 2436±140 | 2458±141 | 21±51 | 0.9±2.1 |
| Left superior frontal gyrus | 2816±134 | 2854±120 | 38±43 | 1.4±1.6 |
| Left superior parietal cortex | 2286±109 | 2319±121 | 33±72 | 1.5±3.3 |
| Left superior temporal gyrus | 2856±120 | 2881±153 | 25±63 | 0.8±2.3 |
| Left supramarginal gyrus | 2580±85 | 2603±78 | 23±36 | 0.9±1.4 |
| Left temporal pole | 3792±272 | 3797±259 | 5±188 | 0.3±4.9 |
| Left transverse temporal cortex | 2223±170 | 2258±176 | 35±88 | 1.6±3.9 |

**Supplementary Table 2. Changes in cortical thickness during ECT treatment are shown for all regions of the Desikan Killiany atlas.** Significant increases in several regions were observed between baseline and post-ECT scans. Most other regions also showed an increase in cortical thickness, which was not significant after Bonferroni correction for testing multiple regions.
Pre-ECT data was calculated as the average of both baseline scans. Only data from subjects with available post-ECT MRI data is shown (n=13). *, significant at p_FWE_≤0.05 (Bonferroni corrected); †, FDR controlled at q*≤0.05 (Benjamini-Hochberg procedure).
